# The lived experiences of women exploring a healthy lifestyle, gestational weight gain and physical activity throughout pregnancy

**DOI:** 10.1111/hex.13514

**Published:** 2022-05-05

**Authors:** Lisa Newson, Kathryn Bould, Bronte Aspin‐Wood, Lauren Sinclair, Zainab Ikramullah, Julie Abayomi

**Affiliations:** ^1^ School of Psychology, Faculty of Health Liverpool John Moores University Liverpool UK; ^2^ Patient and Public Involvement Representative, Member of the Public Serviceuser of Mamafit intervention Liverpool UK; ^3^ School of Applied Health and Social Care, Faculty of Health, Social Care and Medicine Edge Hill University Ormskirk UK

**Keywords:** experiences, gestational weight gain, Grounded Theory, obesity, physical activity, pregnancy, qualitative

## Abstract

**Background:**

Weight gain is inevitable during pregnancy. However, high prepregnancy body mass index and excessive gestational weight gain are associated with poor pregnancy outcomes. Understanding the experiences, social influences and decisions women make to maintain a healthy lifestyle during pregnancy are essential to consider how to improve services and interventions to help women engage in a healthy diet and physical activity (PA) behaviours.

**Objective:**

The study investigated women's opinions and lived experiences of engaging in a healthy diet, promoting optimal gestational weight gain and PA during and after pregnancy.

**Design and Methods:**

Twenty‐two pregnant women contributed to qualitative data collection for this Grounded Theory (GT) study. Nineteen women completed semi‐structured interviews and three patient and public involvement (PPI) representatives sought to validate the analysis and GT framework.

**Results:**

Two substantive categories were constructed: (1) Evolving from ‘I’ to ‘we’, as informed by two subcategories and (2) the power of information and guidance, as informed by three subcategories. These categories informed the core category, ‘A navigational journey and evolution of the pregnant self’. The navigational journey involves constantly searching for knowledge and information to support and balance the interests of personal beliefs, the health of their unborn baby, their social circle and the wider world. A woman's psychological capability (e.g., their knowledge of a healthy lifestyle and confidence to implement such knowledge) is continuously tested.

**Conclusions:**

Pregnancy may create a ‘teachable moment’ but there is a need for appropriate guidance from professionals to assist with lifestyle choices during pregnancy. The findings showed a significant influence of online resources, and lack of guidance on behaviour during pregnancy and may highlight areas of focus for future research and intervention.

**Public Contribution:**

Three pregnant women were recruited to act as PPI representatives to assist with the validation of the analytical findings and aid the final theoretical saturation of the GT framework. Commentary from these PPI representatives was used to validate the analysis and support the interpretation of the data. In addition, these PPI representatives were also invited to provide commentary on the draft manuscript and those involved in this later process have been included as coauthors.

## 1 BACKGROUND

Weight gain is inevitable during pregnancy.^1^ However, high pre‐pregnancy body mass index (BMI) and excessive gestational weight gain (GWG) are associated with poor pregnancy outcomes, such as pre‐eclampsia, gestational diabetes, caesarean section, high blood pressure, congenital disorders and perinatal death.^2^, ^3^, ^4^, ^5^, ^6^ Currently, in the United Kingdom, there are no official clinical guidelines regarding appropriate GWG.^7^ However, the American Institute of Medicine (IOM)^8^ provides specific recommendations on GWG concerning pre‐pregnancy BMI: for example, women classified as healthy weight are advised to gain between 25 and 35 lbs (11.5–16.0 kg); compared to women living with obesity, advised to gain less at 11–20 lbs (5–9 kg) in total. However, evidence,^9^ which has evaluated over one million global pregnancies, found that 47% of women gained more than the IOM recommendations, indicating a high prevalence of excessive GWG and increased risk of complications (regardless of initial BMI).

Despite a lack of UK guidelines regarding appropriate GWG, there are guidelines regarding physical activity (PA), recommending at least 150 min of PA per week during pregnancy.^10^ UK clinical guidelines^11^ also state that all pregnant women should receive advice about healthy eating and PA from midwives. During pregnancy, women are more interested in nutrition, have increased motivation and are more likely to seek advice about their health.^12^, ^13^ However, pregnant women have identified a lack of information and support from healthcare professionals, which is a barrier to maintaining a healthy lifestyle during pregnancy.^14^ In addition, research shows that levels of PA often decrease during pregnancy^15^ or stop altogether.^12^, ^13^, ^16^, ^17^

Literature investigating views of healthcare professionals suggests that midwives do not utilize opportunities to discuss weight management and lifestyle choices^18^ and report a lack of time, knowledge and skills to deliver such information. Moreover, midwives report a lack of confidence in discussing issues related to weight management, specifically in regard to GWG, and therefore conversations about appropriate GWG are often avoided.^19^

Interventions for promoting optimal GWG have varying results, with limited success. For example, researchers^20^, ^21^ have designed and delivered interventions to promote a healthy diet, increased PA and implemented GWG guidance, and found that this positively influenced GWG in their study populations. However, other studies have found that interventions during pregnancy were unsuccessful when attempting to change PA behaviour or reduce excessive GWG,^22^ or that attrition rates from interventions were high.^23^, ^24^, ^25^

Understanding the experiences, social influences and decisions women make to maintain a healthy lifestyle during pregnancy are essential to consider how to improve services and interventions to help women engage in a healthy diet and PA behaviours throughout and post‐pregnancy. However, there is limited research that explores the experiences and perceptions of pregnant women, their diet and PA behaviours. Qualitative methods permit an exploration of experiences and knowledge to understand behaviour and develop new insights.^26^ Specifically, Grounded Theory (GT) is appropriate when there is little known about a phenomenon and focuses on creating conceptual frameworks via an inductive analysis of the data.^27^ The study aimed to investigate women's opinions and lived experiences of participating in a community intervention for women, promoting optimal GWG and PA during and after pregnancy.

## 2 METHODS

### 2.1 Design

The study's primary aim was to construct a theory that offers an understanding from and is connected to the very reality that the theory is derived to explain.^28^ Two philosophical stances influenced this GT study. Firstly, symbolic interactionism^29^ explored the pregnant women's lives and behaviours surrounding diet and PA, and secondly, constructivism, the process of how the women understood their pregnancy and lifestyle advice (meanings) and how that understanding subsequently informed their actions.^27^

### 2.2 Participants and procedure

Recruitment of participants took place at a local lifestyle intervention for pregnant women across Merseyside (England, UK). This intervention aimed to promote optimal GWG and PA during and after pregnancy. Upon the first contact and before attending the intervention itself, the pregnant women were invited (initially via email and then followed up via face‐to‐face meeting) to participate in this study. This study recruitment was an independent process and was neither informed nor influenced by the women's engagement with the intervention itself.

The research inclusion criteria invited women with a BMI greater than 20 kg/m^2^, aged 18 or over, in their third trimester (>27 weeks gestation) and who had a noncomplicated (single) pregnancy. Due to the nature of the research, participants were required to speak English to enable them to communicate their thoughts and beliefs via interview. In addition, women were excluded from participating if deemed a high‐risk pregnancy^11^ (defined as women requiring additional care). The purpose of this study was explained both verbally and in writing, allowing time to consider participation. Participants provided written consent; this included consent to the interviews being audio‐recorded, subsequently transcribed verbatim and selected anonymized quotations to be used as evidence to support the analysis and publication by the research team. Interviews were conducted in a private environment to allow participants to feel comfortable and encourage them to talk freely.^30^

In line with GT methodology, we engaged in both purposeful^31^ and theoretical sampling,^32^ alongside simultaneous data collection and analysis to guide both the sampling and analytical strategies and aid the development and refinement of the categories and theory construction.^33^ Theoretical sampling facilitated variability in the sample of women and supported further exploration of the developing categories and theory formation. In the later stages of theory construction, it was apparent that a greater level of understanding from women carrying their first pregnancy was needed, and therefore these women were targeted for recruitment. In addition, further exploration of the barriers to PA and healthy eating before maternity leave commenced was required; therefore, recruitment became more focused upon women in the earlier stages of pregnancy as identified by the pregnancy intervention service lead.

In total, 22 pregnant women participated. Nineteen women completed digitally recorded semi‐structured one‐to‐one interviews (interviewed by L. S. *n* = 8 and B. A. W., *n* = 11; mean duration: 64 min). Of these, 79% were White British, mean age 24–42 years old, with 62% of women expecting their first child. An initial GT was constructed, and to validate the findings and aid the final theoretical saturation of the GT framework^34^ another three pregnant women acted as patient and public involvement (PPI) representatives and engaged in discussions directed around the analytical findings. These PPI representatives were recruited as per the original recruitment procedure, and we considered these representatives to be heterogeneous in their pregnancy experiences (though not experienced in academic research processes). PPI representative 1 (Rep1) was aged 30, having her first baby at 34.5 weeks gestation (1 previous miscarriage), White British and considered herself fit and healthy; PPI representative 2 (Rep2) was aged 27, having her second baby, currently at 27 weeks gestation (first child now 3 years old), mixed ethnicity White British/Middle Eastern, had experienced hyperemesis previously; PPI representative 3 (Rep3) was aged 35, at 37 weeks gestation of a second pregnancy (first pregnancy through in vitro fertilization delivered stillborn), White British.

### 2.3 Interviews and PPI codevelopment

A semi‐structured interview schedule, developed between the research team, and in consultation with health professionals and pregnant women, explored the participant's perceptions and expectations of their diet and PA during pregnancy. In addition, it aimed to gather opinions on potential challenges to engaging in specific dietary and PA advice (Table 1). Questions in the interview were expanded upon throughout and were not utilized as a strict script; for example, a vital part of the interview process involved listening and following‐up answers to interviewee responses, and in addition, iterations were made to the interview schedule for later interviews so to explore emerging categories during theoretical sampling. Sensitivity to dialogue and linguistic context^35^ was shown in the commitment to avoid leading questions, for example, avoidance of inferring a definition of a healthy lifestyle (asking ‘what does healthy mean to you?’).

**Table 1:** Interview questions and PPI agenda

Interview question	Probes^a^
What does the definition of healthy mean to you?	
– How would you describe your eating and physical activity habits before pregnancy? – Do you feel like your habits have changed during pregnancy?	– Can you tell me a bit more about that? How much and in what way?
– What are your thoughts surrounding physical activity during your pregnancy? – What advice have you been given about participating in physical activity or exercise during pregnancy?	– Healthy eating? – Who gave you or where did you get this information? – Was the advice clear/did you understand the advice? Did you follow it or ignore it? Explain.
– Can you think of ways pregnant women can be physically active?	– Give me a list. Consider all types of activities, including leisure, walking for transportation, work activity, home activities and so forth.
– Were you satisfied with your prepregnancy weight? – How do you feel about gaining weight during pregnancy?	– Tell me more about why you were or were not satisfied with your weight. – Why do you feel like this?
– What should women eat during pregnancy? – Is there anything that keeps you from eating the kinds of foods that you want and need to be healthy? Please explain.	– Shopping, transportation, time constraints for shopping or preparing foods, finances, work or household responsibilities, other children, healthy food choices, not sure? Etc.
– What encouraged you to join this intervention? – Are you happy with the support and guidance you have received from health professionals?	– Tell me more about the advice you received?
*PPI agenda and example from a discussion*	
– Welcome, thank you and introductions – Process and seeking consent – Overview of project – Seeking insight and experiences of individuals – Analytical discussion. Example: *One of the categories describes the women's experiences of encouragement and support to engage in PA. The role of the midwife was considered important but women reported varied advice regarding diet, foods and physical activity—perhaps signposted to PA but not reinforced, mixed messages or misunderstanding of some advice. Diet and PA not prioritized in Midwife role. Do you think these comments/quotes are representative of women's/your experiences? What's happening here? Have we understood this correctly, do you believe our analysis reflects their experiences?* – Consideration for improvement and future work – Summary, thank you and close	

Abbreviations: PA, physical activity; PPI, patient and public involvement.

^a^ Probes were used to get more information from the participants if answers were very short or lacked substance. If participants gave some information but needed to expand, the researcher would use probes such as ‘That's interesting, tell me more’.

The PPI process was conducted as online workshops, for which authors (L. N. and K. B.) presented the findings alongside the GT framework and asked the women to be open in their feedback and reflect upon their sense of meaning, experience and value of the theoretical framework (Table 1).

### 2.4 Data analysis

Interviews were audio‐recorded and transcribed verbatim. Post‐interviews, reflective notes and commentaries regarding the interview process and initial insights from the participants were made, these reflections were entered into the analysis as additional supporting data linked to the transcribed scripts for each participant. Following GT methodology, data collection and inductive analysis were carried out concurrently using a systematic approach of immersion in the data, open and focused coding, memo writing and grouping data into categories while simultaneously collecting further focused data to explore the constructed categories.^32^

Investigator triangulation^36^ was adopted throughout the research process. The research team acknowledged ‘methodological self‐consciousness’ and discussed how their experiences, as clinicians, academics and personal accounts, could have influenced the analysis.^32^, ^37^ The research team was all female, with three authors (J. A., K. B. and L. N.) having children and acknowledged their mixed experience with pregnancy services. It is noteworthy that during the PPI process, refinement of analysis and write‐up process, one author (K. B.) was pregnant and therefore contributed both research and lived experience to her analytical contributions. Two authors (B. A. W. and L. S.) conducted the one‐to‐one interviews, who, at the time, had no direct experience of pregnancy services and remained open to the stories and meanings expressed by the participants during the interview processes. Field notes during, and reflection notes following, the interviews acted as initial memos and aided understanding as analysis commenced. Throughout this process, the research team reflected on the interviewing techniques and considered iterations to the interview schedules in response to the analytical coding, category creation and theory construction.

Upon construction of the provisional GT, the categories and framework were analysed against the context of previous research^38^ (known as ‘sampling the literature’ in GT^34^, ^38^). Finally, the PPI process (conducted by K.  B. and L. N.) sought to aid the theoretical saturation of the GT to invite a further selection of pregnant women to coconstruct and validate the final analysis. These PPI discussions were not digitally recorded although written notes and verbatim quotes were made throughout the process. Following the PPI workshops, the analysis was refined to account for this additional insight (by K. B. and L. N.). In line with best practice for PPI, to promote transparency and partnership working, the PPI representatives were each invited to co‐produce the final analysis; to support the discussion, particularly in regard to the implications for practice and to provide additional commentary and feedback on the draft of this manuscript. For those involved in this process, they have been included as co‐author (Z. I.).

### 2.5 Ethics and data availability

Ethical approval was granted by the NHS Health Research Authority (ID 242804; REC reference 17/YH/0132). To promote transparency in context,^39^ the findings section of this manuscript presents verbatim quotes from a range of the participants to act as evidence to support the analytical commentary. In recognition of legal and ethical processes, participants of this study did not agree that their transcripts were fully shared publicly, so supporting data beyond the sample quotation extracts is not available.

## 3 FINDINGS

**Figure 1:**
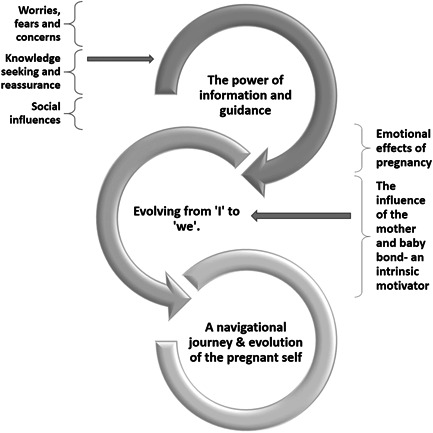
Analytical model to represent the interplay between analytical categories.

The present study explored motivations and expectations during pregnancy concerning PA, dietary behaviours and GWG. By adopting a GT approach,^27^, ^32^, ^40^ the research has highlighted a range of beliefs that impact women's intentions to practice a healthy diet and maintain PA throughout their pregnancy. Two substantive categories were constructed from the data: (1) evolving from ‘I’ to ‘we’, as informed by two subcategories and (2) the power of information and guidance, as informed by three subcategories. These categories informed the core category: A navigational journey and evolution of the pregnant self (see Figure 1).

The core category expresses the relationships among categories and reflects pregnant women's social, psychological and behavioural processes in practising a healthy diet and maintaining PA throughout their pregnancy. Moreover, the core category demonstrates continued development throughout pregnancy. All the categories in the GT framework contributed to pregnant women's experiences and expectations, focusing on their GWG, dietary and PA expectations and their motivations for joining the lifestyle intervention. This model helps to represent how although the substantive categories are distinct, they are not isolated as they interact and inform each other.

### 3.1 Substantive category—Evolving from ‘I’ to ‘we’

A key motivator for behaviour change during pregnancy was the identification of ‘being pregnant’ and the formation of a maternal bond. A further influence on motivations, expectations and the evolving pregnant self was the impact of the changes experienced by the mother‐to‐be, particularly in relation to the transition from thinking about herself and her unborn child.

*Moving from I to we, I definitely thought of that, I was aware, and I wanted a balanced diet*. PPI REP 1

This substantive category portrays the impact of emotional and physiological changes during pregnancy, the mother's attachment to the foetus and the roles that these factors have in manipulating lifestyle behaviours.

#### 3.1.1 The influence of the mother and baby bond—An intrinsic motivator

While evolving into their pregnant selves, mothers became increasingly aware of their baby's development, had a strong sense of responsibility to ensure the health of the unborn child and were actively motivated by the influence of their lifestyle decisions on their baby's health.

*When you think that it is your health and your baby's health that is a very strong motivation you don't need something more*. Interview 3

This motivator was present in all interviews and was reported to impact the uptake of positive health behaviours, such as changes to dietary choices—for example, adherence to dietary guidelines such as the avoidance of ‘unsafe’ foods throughout pregnancy.

*…During pregnancy, I think it is even if you weren't healthy before or not as healthy with your eating beforehand I think it's important to find a concentrated effort during pregnancy*. Interview 17

Knowing that their bodies were looking after their unborn child, women sought increased knowledge and became more aware of diet and food, especially recommended daily nutrient intakes.

*I've bought cereal, you know, with the added folic acid and B12 and things like that. I am more aware of things when I'm buying them, so that has probably been different to before*. Interview 12

The participants wanted to ensure their unborn baby was getting the building blocks for their development and felt that diet was important.

*I think you have to take yourself out the equation and what you want because it's not all about you…and, I'm a person, I'm responsible and want what's best for my child so if that means standing up and cooking when I've just got home from an 11 and a half shift, so be it, because it's important*. Interview 10

As a result, the baby's health became a motivational factor, many women expressed the desire to improve their daily lifestyle and health behaviours.

It is noteworthy that some women felt disconnected from their old selves because of their pregnancy's evolution, and their body was no longer just ‘theirs’. In addition, emotional wellbeing was affected by the inevitable ever‐changing body due to pregnancy.

*I need to keep my identity – want to retain a bit of me, and keep active, because I'm not just a vessel*. PPI REP 3

The women who were able to engage in PA throughout pregnancy acknowledged the positive influence of attending PA classes in reconnecting with oneself, demonstrating that persevering with PA participation can benefit emotional wellbeing. However, there was little acknowledgement of the wider health benefits of PA to women or their babies.

#### 3.1.2 Emotional effects of pregnancy

Several women acknowledged their reluctance to engage in PA due to lack of knowledge and perceived potential harm to the baby^41^. In addition, a description of physical separation between the ‘baby’ and ‘me’ was observed; the women were motivated to avoid behaviours that may negatively impact the baby's development. It was also apparent that for many women, PA was considered a mixed behaviour, with the women especially acknowledging the emotional health benefits of PA to themselves, but that PA could be considered a harmful behaviour to engage with for the baby's development and as such this created anxiety and influenced their decision for ceasing or reducing PA engagement.

*I just couldn't; just not something that could be unsafe or harm the baby; you know, like put stress on the baby just because I weren't aware; and all just because it makes me feel better about myself*. Interview 3

Participants described the negative influence of self‐consciousness upon adherence to PA due to negative self‐images of being pregnant around others.

*Women feel like self‐conscious, or, when they go the gym, others wondering if they're just overweight rather than actually pregnant*. Interview 11

Further comments also discussed how worries around self‐image could increase during pregnancy, especially if women compared themselves to others. In addition to a lack of self‐confidence, most women discussed the unease they felt in the face of losing control over their bodies and their worries about excessive GWG.

*Am I going to gain back control of my body? Am I going to be able to sustain not going any bigger than what I am*. Interview 10

### 3.2 Substantive category—The power of information and guidance

This substantive category explored the impact of knowledge and understanding, or lack of, on positive behaviour and lifestyle choices throughout pregnancy. The health of their unborn child influenced an active search for information regarding lifestyle during pregnancy, and the internet was a vital resource and a significant part of the pregnancy journey. However, the power of healthcare professionals to discuss or raise concerns about issues such as GWG leads to some women being uninformed or ill‐equipped.

#### 3.2.1 Knowledge‐seeking and reassurance

Women sought credible information and expressed a desire for knowledge to facilitate behaviour change surrounding diet and PA. All women had some basic general knowledge surrounding diet and PA in pregnancy. However, this knowledge did not automatically compute their ideals for their pregnant self and positive, healthy behaviour. All women recalled foetal health motivating active seeking of information to guide their dietary and PA decisions once learning of their pregnancy, and for some, this search for information enhanced the women's ability to make healthy lifestyle choices.

Advice was sought from the midwife, whose knowledge was held in high regard. Women sought credible information and expressed a desire for knowledge to facilitate behaviour change surrounding diet and PA. Throughout this study, a key behaviour observed was the act of knowledge‐seeking and the expectation for advice to be provided by midwives.

*I was thinking right no just get to my 12 weeks, I'll see the midwife, and they'll sort me out*. Interview 1

However, in line with previous research,^1^ many women expressed disappointment in midwives due to inconsistent guidance.

*Don't think midwife has brought it up, anything about diet or PA to be fair*. PPI REP 1

Participants in this study were explicit in suggesting that midwives did not discuss the relevance or importance of weight gain during pregnancy.^1^, ^19^ During their first antenatal appointment, midwives recorded the women's weight as part of routine care. This was the only time weight was mentioned by the midwife, and the participants are unanimous in the perception that there was no discussion of the importance of weight gain or GWG averages. The lack of discussion influenced the participants' beliefs surrounding GWG:

*No, like the midwife's not really said anything so about my weight gain, which I would presume is probably a good thing. Because if she was worried she would bring up*. Interview 14

Participants felt it was the responsibility of the midwife to highlight any issues with GWG. This led participants to believe that GWG was not significant or that the participant's own GWG was healthy.

*But I haven't really mentioned it to anybody, including my midwife; I just thought if there was an issue or she thought there was an issue, then she would bring it up*. Interview 17

The lack of discussion with the midwife about weight gain reduced the overall perception of its significance. Some participants believed that if GWG were a subject of consequence during pregnancy, the midwife would monitor or mention it:

*… And then since then they've not weighed me, but they've obviously measured the baby. So I feel like they're not that bothered about what I weigh, it's more about what the baby weighs*. Interview 14

Appointments with the midwife are an important part of maintaining a healthy pregnancy, and participants felt that if GWG were relevant, the midwife would discuss it.^42^

*Midwife told me not to weigh [myself], I was shut down*. PPPI REP 3

The lack of information further cemented concerns and influenced the avoidance of PA. In the absence of informative interactions with midwives, women turned their attention to other sources, such as the internet and apps^43^, ^44^

*all knowledge is googled. Would be good for a midwife to discuss in some way*. PPI REP 1

The internet was considered a useful tool for participants to quickly access information, which often provided reassurance.^45^

*I don't know what I would do without Google. Yeah, I don't know what women used to do [to get information], like all the stuff I've learnt about safe healthy foods*. Interview 12

However, some women acknowledged the dangers of utilizing the internet for acquiring information and guidance, due to the ambiguity of the information provided and the motivations of the sources. Several participants acknowledged a lack of available reputable dietary and PA information.^46^

*There is advice and general information on them, but it's obviously trying to get you to think about their products, buy their products*. Interview 1

Nearly, all women reported a reduction or cessation of PA during pregnancy. This reduction in regular PA was attributed to a lack of knowledge about the safety of such activities and limited opportunity to ask questions or seek guidance about PA.

*Because I've actually looked for quite a few of these [type of exercise classes]… There's not many accessible Pilates or yoga for beginners classes out there or even stretching that are definitely safe during pregnancy*. Interview 15

Although participants reported a decrease in PA, they expressed an interest in low‐intensity exercise. However, limited availability and knowledge of classes were frustrating for the women.

*I was looking myself for some Pilates classes, and I haven't been able to find any locally*. Interview 17

The difficulty of engaging in high‐intensity activities alongside pregnancy symptoms was often present, and the limited availability of low‐intensity PA classes meant PA levels were reduced for many women. This reflects the need for more formal guidance for safe PA during pregnancy and indicates, especially regarding low‐intensity activity, the need for more availability of pregnancy lifestyle interventions.

*I've definitely made more of a conscious effort to do more walking because I felt like that has definitely helped my back, and being a bit more mobile just generally with aches and pains it's helped. Also, I'm going more regularly to Yoga… And again, that's just like because it's making me feel better*. Interview 14

#### 3.2.2 Social influences

External factors influence behaviours surrounding diet and PA throughout pregnancy. Friends and family influenced the women's dietary and PA behaviours significantly.

*People around me eat non‐stop, say don't do activity, don't to that, don't push pram, pressure from other directions*. PPI REP 2

Social influences often encouraged engagement in healthy eating and PA to pursue a healthy lifestyle and healthy pregnancy. However, negative comments from others would incite worry and panic.

*Couldn't argue with them because I didn't know myself whether it's right or wrong, so I listened and avoiding it*. Interview 8

However, negative comments from others also motivated the women to seek information and, when able, to join the lifestyle intervention, as women strived for further information and wished to be with other pregnant women. The social pull of the lifestyle intervention was reported as a key motivator for many of the participants.

*Feel a little bit more comfortable because you are with other people that are pregnant who often have the same questions and concerns as I do*. Interview 1

Women felt pressure from others to keep weight gain within a healthy range. However, several participants were passive in discussions and viewed weight gain as part of pregnancy. This concurs with our earlier finding—‘weight gain is inevitable’.^1^

*I know a lot of women who would probably take that to heart, but I'm pregnant, I'm having a baby, I'm growing a baby*. Interview 10

#### 3.2.3 Worries, fears and concerns

A key consequence of the absence of available reliable information surrounding knowledge growth during a women's pregnancy is the manifestation of worry and concern. This lack of knowledge feeds into the worry of self‐blame; therefore, this results in the avoidance of PA as there is a lack of knowledge surrounding potential consequences.

*Couple of my friends and stuff have had miscarriages and things. I just didn't want to risk it.* Interview 11

Similar to findings from previous research, fear of harming the baby, and restricting engagement in PA, stemmed from the womens uncertainty regarding possible negative consequences for their unborn child^47^, ^48^, ^49^ and participants felt that engaging in PA could put their unborn child at risk, creating a need for professional guidance to reassure them.

*I wasn't sure what [exercise] you can and can't do and if anything is bad [for the baby]*. Interview 19

These feelings arose from the lack of guidance received on the safety of PA during pregnancy:

*No one has actively told me anything [about the safety of physical activity] if you know what I mean, but myself I've gone out [to the lifestyle intervention] to get that information*. Interview 19

Inconsistent information can elicit worry and concern, which was evident throughout the study, especially concerning GWG and participation in PA.

*What do you do when your midwife tells you something, or worse, tells you nothing at all, and the internet says something different. You can't help but worry*. Interview 16

Prior knowledge of and engagement in PA did not necessarily reduce concerns. Participants who were active before pregnancy often did not want to continue until they were sure it was safe. This was one of the motivations regularly mentioned for attending the lifestyle intervention. Participants who had access to the lifestyle intervention felt reassured by the guidance provided, a key finding in this current study. This encouraged them to continue or begin exercising, whereas, in previous studies, women did not engage in PA due to fears of harming their unborn baby.^47^, ^48^, ^49^, ^50^

Comments inferred that pregnant women would continue PA in pregnancy if knowledge of its positive influence was increased. However, they currently lack trust in their abilities and the knowledge held by others. The majority felt that the risk of engaging in PA was too high, and further guidance was needed, especially considering the health of their unborn child was a priority during pregnancy.

*I'm not going to any classes…I go to the council gyms and…like not to be like disrespectful to them, but I don't get the impression that they would tell you if you were doing something wrong*. Interview 9

### 3.3 Core category—A navigational journey and evolution of the pregnant self

**Figure 2:**
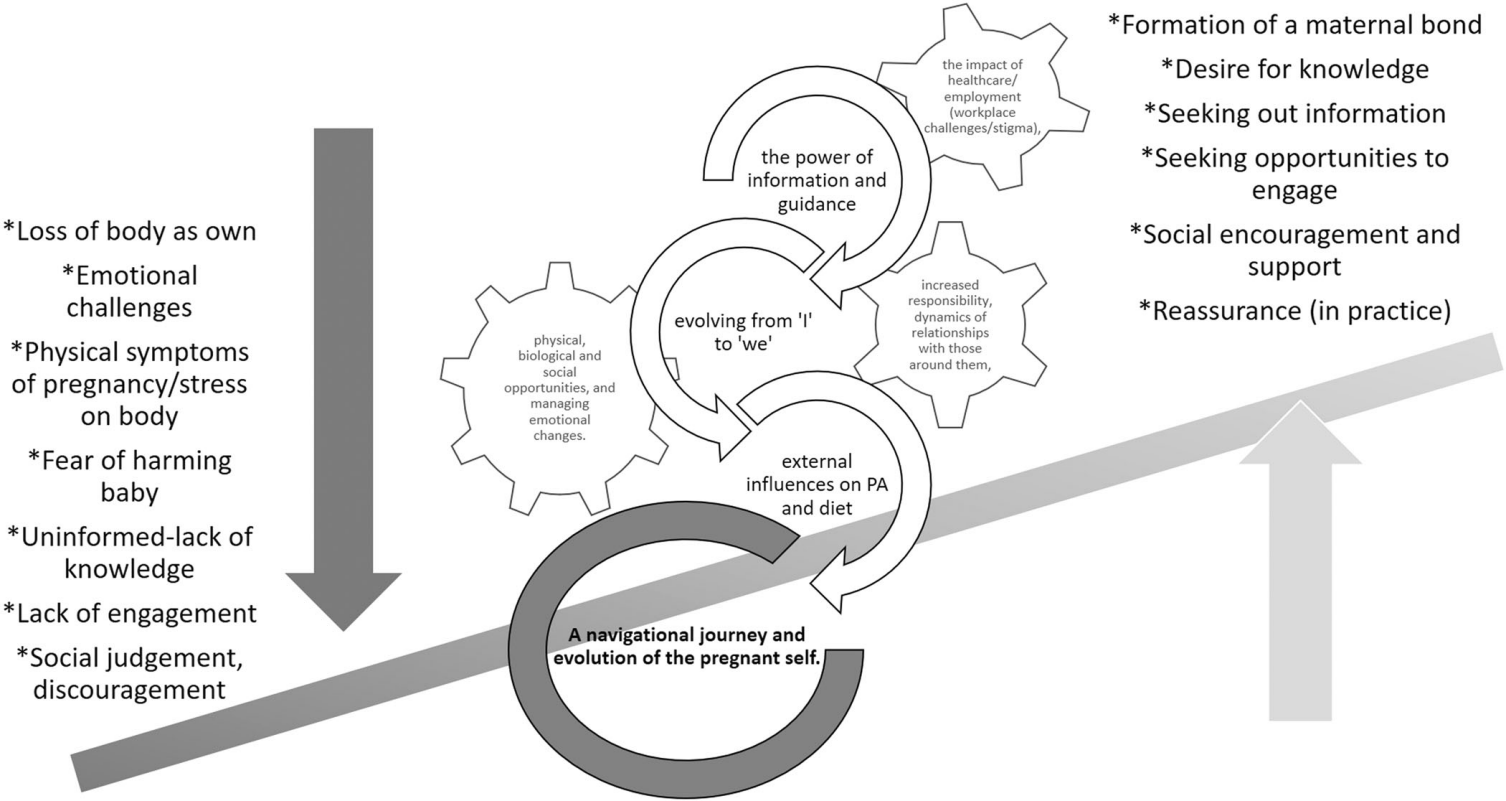
A grounded theory model unpicking the navigational journal and evolution of the pregnant self.

The navigational journey of pregnancy (see Figure 2) involves constantly searching for knowledge and information to support and balance the interests of personal beliefs, the health of their unborn baby, their social circle and the wider world. Many substantial changes are experienced during pregnancy, including increased responsibility, dynamics of relationships with those around them, the impact of employment (workplace challenges/stigma), physical, biological and social opportunities and managing emotional changes. Managing these multiple factors can sometimes be conflicting and may create feelings of isolation for the pregnant woman (for example, when needing or choosing to prioritise pregnancy appointments above work commitments). In the context of behaviour change,^51^ this core category describes that the pregnant woman's psychological capability (e.g., their knowledge of a healthy lifestyle during pregnancy and confidence to implement such knowledge) is continuously tested, and the women learn to manage this. Seeking out information reinforces the capability and motivation to engage in positive lifestyle behaviours. Whereas limited information or discouragement from significant others to engage in lifestyle behaviours (e.g., PA) reduces motivation and opportunity. Women need social support from services, work and family members to engage in optimum health decisions. Pregnancy, in this context, is an ongoing period of change, and to ensure motivation to engage in behavioural and lifestyle interventions, women, must be actively supported throughout pregnancy, and given an opportunity (physical and emotional) to seek optimum health for themselves and their unborn child.

## 4 DISCUSSION

Previous research utilizing qualitative methods of women's experiences typically focuses on the earlier stages of pregnancy^52^, ^53^ or postpartum^54^; few studies explore the lived experiences of pregnant women approaching or in their third pregnancy trimester. However, GWG, diet and lifestyle behaviours are important to monitor and promote throughout all stages of pregnancy. Given that, evidence suggests the timing of GWG influences both maternal and perinatal outcomes^55^ hence this study adds to our understanding of women's whole pregnancy experience. The findings in this study indicate that women have a desire to engage in healthy behaviours to support foetal health and this sense of responsibility created an awareness of diet and a desire to seek out information. However, information on diet and PA as a standalone resource is not always sufficient to encourage behaviour change. Further guidance is needed to assist, inform, implement and reassure women throughout the pregnancy journey.

This study indicates that not receiving information on GWG from a midwife or healthcare professional changed beliefs about GWG, even when participants had prior knowledge from other sources. Currently, in the United Kingdom, GWG is not monitored throughout pregnancy and therefore too much or too little GWG may be more difficult to recognize.^56^ This is problematic if excessive GWG goes unnoticed by the midwife, especially if women are concerned about their own weight gain but feel it is the responsibility of the midwife to highlight any issues. Missed opportunities to discuss or improve women's GWG could have serious health implications and therefore these issues need to be addressed. Previous research indicates a lack of discussion with the midwife regarding GWG as midwives find it a sensitive topic^57^ and often avoid talking about it with patients.^19^, ^58^ To avoid missed opportunities, weight gain should be discussed and monitored by the midwife for all pregnancies. As identified in this study, the pregnant women expected and wanted their health professional (specifically their midwife) to raise the issue of GWG relevant to both the babies' health development and their own health status. However, further research is needed to identify the best methods of integrating discussions and monitoring of GWG into routine care.

General dietary awareness and engagement in healthy eating behaviours were common among participants. Participants described making healthy nutritional choices and aimed to maintain the health of the foetus through diet. In previous research, it has been suggested that some health events or life changes such as pregnancy may encourage individuals to implement healthier, risk‐reducing behaviours.^59^ In the present study, pregnancy increased participants' awareness of their diet providing support for pregnancy as a teachable moment.^60^ Foetal health motivated participants to seek information about diet and PA during pregnancy, especially from online resources. Due to the easy access and seemingly limitless information, the internet was a quick and easy way to answer queries and aid healthy choices. Past research shows that the internet is a common source of information during pregnancy^43^, ^44^ and may positively influence behaviour,^45^ and the present findings support this. Although the women in this study did not provide detail on the information for which they accessed, previous research indicates that online resources can be unreliable^61^ and not in accordance with UK pregnancy guidelines.^62^, ^63^ In light of this, it might be beneficial for healthcare professionals to consider guiding women to reliable sources of online information.

However, the women in this study were clear that information only was not sufficient as standalone support for maintaining a healthy lifestyle during pregnancy. Similar to previous research, fear of harming the unborn baby was a barrier to PA^47^ and participants felt PA put the foetus at risk, creating a need for professional guidance to reassure them on an ongoing basis. A key finding of the present research was that the participants, who had access to the lifestyle intervention, felt reassured by the feedback and physical guidance provided. This encouraged them to continue or begin exercising, whereas in previous studies women simply did not engage in PA due to fears of harming their unborn baby.^49^, ^50^ These findings highlight a need for healthcare providers to review the lack of support around PA and offer guidance that addresses the assumptions about PA being dangerous.

Future research could test the feasibility, applicability and acceptability, from a professional and patient point of view, of merging routine care and online support. Innovative interventions are needed to help women to make healthy lifestyle choices during pregnancy. Using the findings from this study, future interventions may consider implementing supportive guidance on lifestyle topics and professional support for PA. Highlighting the importance of diet, GWG and PA for the health of the baby may also influence behavioural choices.

A key strength within this study was the acquirement of data through the thorough process of analysis guided by GT.^32^ Qualitative interviews were conducted with a range of pregnant women recruited within an area of mixed (including high) deprivation, and the interview schedules were reviewed and expanded throughout the research process. This resulted in substantial amounts of data being collected and the formation of strong categories leading to the construction of new knowledge. Moreover, to our knowledge, this is the first study that has sought to validate theoretical analysis through PPI involvement.

The findings may be transferred to other pregnancy settings and offer recommendations for improvements to healthcare services. However, we acknowledge that the women recruited into this study, who had made contact with a lifestyle intervention may have been more motivated and knowledgeable regarding a healthy lifestyle during pregnancy. This may suggest that some women are less motivated and may possibly experience additional challenges to engage in PA and healthy lifestyle behaviours throughout pregnancy. Further expansion of recruitment could be employed for pregnant women who do not attend antenatal appointments or engage in pregnancy behavioural interventions. In addition, it is noteworthy that the participant sample was not diverse in terms of ethnicity, but deemed representative of the local area, the sample was mostly White. It is therefore important to consider the inequality in healthcare for women from Black or Minority Ethnic groups^64^, ^65^ who may have perceived their access to care and pregnancy experience, differently from the findings presented.

## 5 CONCLUSIONS

This study explored the capabilities, motivations and opportunities of pregnant women to achieve optimum diet and PA behaviours throughout their pregnancies. Findings demonstrate that women are highly motivated to engage with positive behavioural lifestyle changes throughout pregnancy but are hindered by various negative influences, including a lack of information and reassurance from health professionals. Future interventions may benefit from addressing these barriers and considering the significance of symptom management and foetal health in facilitating behavioural choices during pregnancy. Online resources should be considered as a part of routine care and healthcare organizations may consider revising current guidelines for midwives and pregnant women regarding GWG.

## AUTHOR CONTRIBUTIONS

**Lisa Newson**: Conceptualization, methodology, resources, recruitment of participant strategy, validation, formal analysis, patient and public involvement (PPI) data curation, writing – original, writing – review and editing, visualization, supervision. **Kathryn Bould**: Formal analysis, PPI data curation, writing – review and editing, visualization, supervision. **Bronte Aspin‐Wood**: Conceptualization, methodology, recruitment of participants, data curation, formal analysis, writing – original. **Lauren Sinclair**: Conceptualization, methodology, recruitment of participants, data curation, formal analysis, and writing – original. **Zainab Ikramullah**: Analysis, visualization, writing – review and editing. **Julie Abayomi**: Conceptualization, recruitment of participant strategy, validation, writing – review and editing, visualization.

## CONFLICT OF INTEREST

The authors declare no conflict of interest.

## ETHICS STATEMENT

Ethical approval was granted by the NHS Health Research Authority (ID 242804; REC reference 17/YH/0132). To promote transparency in context, the findings section of this manuscript presents verbatim quotes from a range of the participants to act as evidence to support the analytical commentary. In recognition of legal and ethical processes, participants of this study did not agree that their transcripts were fully shared publicly, so supporting data beyond the sample quotation extracts is not available.

## Data Availability

Raw data has been included as evidence via extracted quotes from verbatim transcripts as samples of evidence. Full transcript release has not received ethical approval or participant consent. For further study details, please contact the corresponding authors. The authors confirm that the data supporting the findings of this study are available within the article.
